# A Phosphite Dehydrogenase Variant with Promiscuous Access to Nicotinamide Cofactor Pools Sustains Fast Phosphite-Dependent Growth of Transplastomic *Chlamydomonas reinhardtii*

**DOI:** 10.3390/plants9040473

**Published:** 2020-04-08

**Authors:** Edoardo Cutolo, Matteo Tosoni, Simone Barera, Luis Herrera-Estrella, Luca Dall’Osto, Roberto Bassi

**Affiliations:** 1 Laboratory of Photosynthesis and Bioenergy, Department of Biotechnology, University of Verona, Strada le Grazie 15, 37134 Verona, Italy; edoardoandrea.cutolo@univr.it (E.C.); Matteo.tosoni@studenti.univr.it (M.T.); Simone.barera@univr.it (S.B.); luca.dallosto@univr.it (L.D.); 2 Laboratorio Nacional de Genómica para la Biodiversidad (UGA) Cinvestav, 36821 Irapuato, Guanajuato, Mexico; luis.herrera-estrella@ttu.edu; 3 Institute of Genomics for Crop Abiotic Stress Tolerance, Department of Plant and Soil Sciences, Texas Tech University, Box 42122, Lubbock, TX 79409, USA

**Keywords:** phosphite dehydrogenase (PTXD), *Chlamydomonas reinhardtii*, chloroplast genome engineering, algal cultivation, culture contamination prevention, nicotinamide adenine dinucleotide, site-directed mutagenesis, phosphorous metabolism

## Abstract

Heterologous expression of the NAD^+^-dependent phosphite dehydrogenase (PTXD) bacterial enzyme from *Pseudomonas stutzerii* enables selective growth of transgenic organisms by using phosphite as sole phosphorous source. Combining phosphite fertilization with nuclear expression of the *ptxD* transgene was shown to be an alternative to herbicides in controlling weeds and contamination of algal cultures. Chloroplast expression of *ptxD* in *Chlamydomonas reinhardtii* was proposed as an environmentally friendly alternative to antibiotic resistance genes for plastid transformation. However, PTXD activity in the chloroplast is low, possibly due to the low NAD^+^/NADP^+^ ratio, limiting the efficiency of phosphite assimilation. We addressed the intrinsic constraints of the PTXD activity in the chloroplast and improved its catalytic efficiency in vivo via rational mutagenesis of key residues involved in cofactor binding. Transplastomic lines carrying a mutagenized PTXD version promiscuously used NADP^+^ and NAD^+^ for converting phosphite into phosphate and grew faster compared to those expressing the wild type protein. The modified PTXD enzyme also enabled faster and reproducible selection of transplastomic colonies by directly plating on phosphite-containing medium. These results allow using phosphite as selective agent for chloroplast transformation and for controlling biological contaminants when expressing heterologous proteins in algal chloroplasts, without compromising on culture performance.

## 1. Introduction

Chloroplast transformation represents a powerful tool to introduce transgenes into the genome of the photosynthetic semi-autonomous organelle for heterologous protein expression. Microalgae such as *Chlamydomonas reinhardtii* (hereafter referred to as *C. reinhardtii)* hold a tremendous potential for becoming the preferential host species for the production of valuable bioactive compounds and recombinant proteins directly in the plastid [1]. The major advantages offered by *C. reinhardtii* as a biofactory lie in its unicellular, photoautotrophic lifestyle and the ease of transforming its chloroplast genome via homologous recombination-based approaches [2]. The single chloroplast of the alga represents its largest subcellular compartment and an ideal storage site for recombinant products. Being the *C. reinhardtii* plastid genome (hereafter referred to as plastome) polyploid [3], multiple transgene copies are present in a transplastomic algal cell, boosting yield in recombinant proteins. In contrast to nuclear transgenesis, where DNA insertion occurs randomly in the genome [4], sequences can be targeted to defined chloroplast loci [5]. Moreover, the plastid genome does not seem to be subjected to epigenetic effects and, therefore, does not entail silencing on transgene expression, as it occurs in the nucleus [6].

A number of technical advances have recently been made in the field of chloroplast genetic engineering of *C. reinhardtii*, resulting in an expanded toolkit of transformation vectors and selection strategies, truly projecting this species to the forefront of applied research efforts for the development of cost-effective production platforms of recombinant products. This is testified by the number of recent reviews covering the plethora of recombinant products that have been expressed in this species [7,8]. However, a major obstacle still hinders the industrial-scale cultivation of *C. reinhardtii*, which is related to the risk of culture contamination by parasitic species [9]. This issue concerns open-air cultivation systems, such as raceway ponds, as well as closed systems (photobioreactors). Microbiological pollutants encompass different taxa including zooplankton, microscopic protozoa, rotifers and microscopic crustaceans [10,11], but closed cultivation systems are typically affected by bacteria, including natural symbiont species [12], fungi and viruses. Allelopathic and out-performing microalgae can also limit the growth of species of interest by secreting toxic metabolites and competing for nutrients [13]. Sterility is thus mandatory in closed cultivation systems, while its dispensability would be a tremendously desirable feature. In this respect, a recently developed biotechnological breakthrough involves the genetic engineering of microalgae to thrive in non-sterile conditions by exploiting the selective metabolism of an essential nutrient. This effective, and very attractive, solution is offered by the phosphite dehydrogenase (*ptxD)* transgene, encoding a NAD^+^-dependent oxido-reductase from the soil bacterium *Pseudomonas stutzerii* WM88 [14]. This enzyme catalyzes the conversion of phosphite (PO_3_^3-^), a non-assimilable phosphorous compound, into the metabolically essential phosphate ion (PO_4_^3-^). Since most parasitic organisms cannot use phosphite in their metabolism, the expression of a PTXD transgene in the nucleus [15] was shown to enable the selective growth of *C. reinhardtii* in the presence of phosphite as the sole phosphorous source, effectively preventing the risk of culture contamination by other species competing for available resources. More recently, PTXD was successfully expressed as a plastid-targeted transgene in *C. reinhardtii* [16,17], emerging as a novel selectable marker for the genetic transformation of the plastome [18]. Despite being an attractive system for the chloroplast transformation of *C. reinhardtii*, the use of PTXD as a selectable marker suffers from some intrinsic limitations that hinder its value. Indeed, PTXD-based selection displays a far lower efficiency compared to traditional antibiotic resistance-based methods [18], mainly due to a long selection time (reported up to 60 days), which reduces the recovery efficiency of true transformation events.

The activity of the PTXD enzyme could be theoretically enhanced via rational approaches that alter its catalytic properties by overcoming the factors that may restrict its functionality. Previous in vitro works have sought to modify the enzyme by means of rational mutagenesis and direct evolution, mainly focusing on thermal stability [19] and cofactor binding properties [20]. Based on a previous report [20], we mutagenized two adjacent amino acids located in the cofactor-binding pocket of the enzyme with the aim of relaxing PTXD selectivity towards the nicotinamide adenine cofactors, rendering the enzyme equally functional when using NAD^+^ or NADP^+^ and thus increasing its catalytic efficiency in vivo in the algal chloroplast stromal compartment. Here, we report the effect of expressing an optimized version of the PTXD enzyme in the chloroplast which bypassed the physiological limitations imposed by the chloroplast metabolism, making it suited for efficient function in this NADP^+^-rich environment.

## 2. Results

### 2.1. Site-Directed Mutagenesis of PTXD Cofactor-Binding Amino Acidic Residues

**Figure 1:**
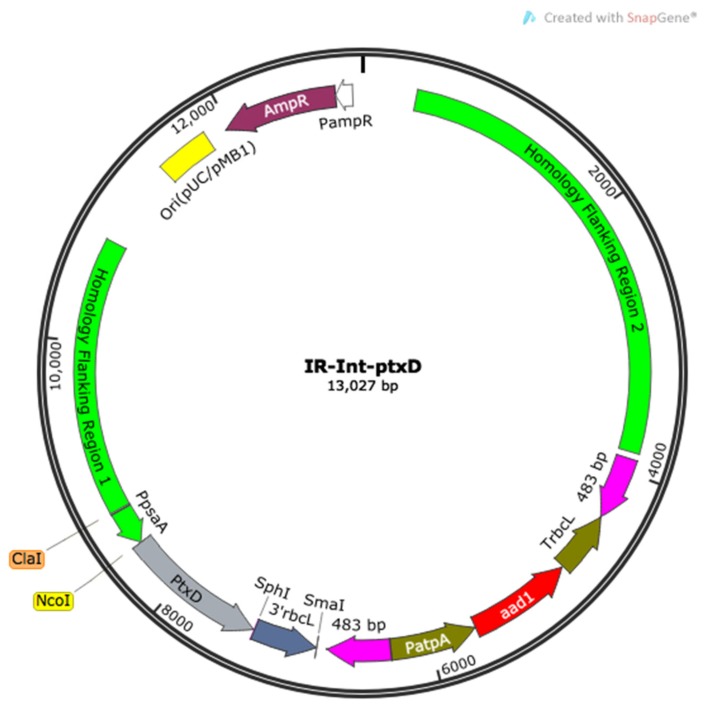
Map of the assembled IR-Int vector carrying the phosphite dehydrogenase (*ptxD*) transgene that was used in this work to transform the plastome of the wild type T222 strain. This vector contains two flanking regions required to enable the homologous-recombination based transgene insertion at the level of the inverted repeats of the chloroplast genome of the alga and the recyclable selectable marker gene *aadA* [22], providing resistance against the antibiotic spectinomycin. The PTXD gene was optimized according to the AT-rich codon bias of the chloroplast genome of *C. reinhardtii* and placed under the transcriptional control of the cis-acting elements pair *psaA* promoter and *rbcL* 3′ untranslated region (UTR) and terminator via a subcloning step through the AtpB-int vector. A mutagenized version of the PTXD enzyme, carrying the substitution of the two adjacent residues Glu-175 and Ala-176 with Ala and Arg, respectively, was produced via site-directed mutagenesis and introduced in the same vector configuration (hereinafter referred to as *ptxD*_E175A-A176R_).

Previous in vitro studies [20] showed that two adjacent residues in the PTXD enzyme, including a negatively charged glutamate, are involved in the selective binding of the NAD^+^ cofactor. The combined substitution of glutamic acid 175 and alanine-176 in PTXD by alanine and arginine residues, respectively, resulted in a relaxed cofactor binding selectivity, enabling the concomitant use of NAD^+^ and NADP^+^. We thus proceeded to mutagenize these two amino acid residues starting from a PTXD template sequence optimized for chloroplast codon usage via a PCR-based, site-directed mutagenesis protocol. By employing a mismatch-containing primer pair, we achieved the desired double amino acid substitution in the PTXD coding sequence and proceeded to introduce both wild type and the mutagenized PTXD versions into a wild type *C. reinhardtii* (T222) strain using the IR-int transformation vector [21] (Figure 1).

### 2.2. The Mutagenized PTXD Version Enables Faster Selective Growth of Transplastomic Lines

The assembled IR-int vectors containing the wild type and mutagenized PTXD version carrying the double amino acid substitution (IR-Int-*ptxD_E175A-A176R_* construct) were subsequently used to transform the chloroplast genome of T222 *C. reinhardtii* cells via the biolistic method [23]. Approximately one week after the bombardment, a number of spectinomycin-resistant colonies (>30) appeared on the selective plates containing the algae transformed with both construct versions. At least 10 independent transformants for each construct were selected and further sub-cultivated on TAP-agar plates in the presence of spectinomycin to increase the transgene copies until near-homoplasmy (6 rounds). Transplastomic lines carrying the mutagenized PTXD version hereafter will be referred to as TP *ptx*D Opt. A transgenic line containing a nuclear insertion of a transgene encoding the WT PTXD enzyme (hereafter referred to as N *ptxD* WT) was used as reference strain for the selective growth experiment performed in this work, as previously reported [15].

**Figure 2:**
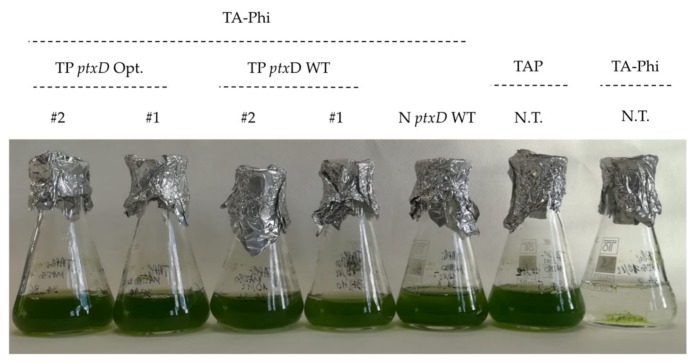
Growth of selected transformant lines in TA-Phi (phosphite-supplemented) medium. Two independent transplatomic lines carrying the wild type PTXD (TP ptxD WT) and mutagenized (TP ptxD Opt) versions, respectively, were included, along with the nuclear transformant (N ptxD WT) and the untransformed T222 wild type. This latter was also cultivated in TAP (phosphate-supplemented) as positive control.

**Figure 3:**
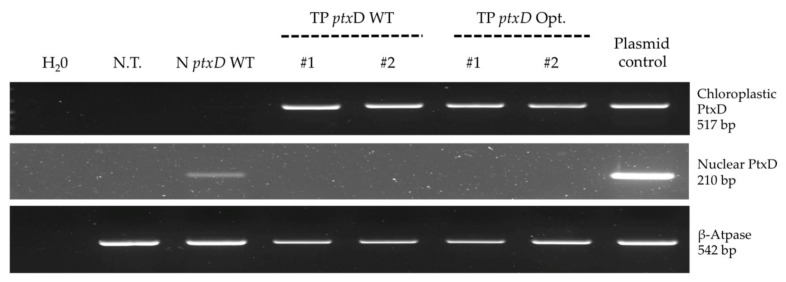
PCR-based genetic characterization of selected transformant lines. Three independent reactions were set up to reveal the presence of the *ptxD* transgene sequence(s). The upper panel refers to the amplification of the chloroplast codon usage-optimized *ptxD* sequence, while the middle panel refers to the amplification of the nucleus codon usage-optimized *ptxD* version. A control reaction (lower panel) was set-up to verify the quality of extracted genomic DNA targeting the ATP-ase β subunit gene. Additional control reactions were performed with appropriate primer pairs on vectors containing the *ptxD* sequences introduced in the algae (IR-int and pChlamy-4 for the chloroplast and nuclear version respectively).

Two transplastomic lines and the nuclear transformant line were further analyzed for the ability to selectively grow in liquid medium supplemented with phosphite as sole phosphorous source. We observed that transplastomic lines carrying the mutagenized PTXD version grew in TA-Phi medium at a similar rate as the nuclear counterpart. In contrast, the transplastomic lines expressing the wild type PTXD version (hereafter referred to as TP *ptx*D WT) grew at a far slower rate in the selective medium. The ability to metabolize phosphite and to convert it into the assimilable form phosphate is strictly dependent on the expression of the PTXD enzyme, since the untransformed T222 line did not grow either in TA (phosphorous-devoid) or in TA-Phi (phosphite-supplemented) medium (Figure 2). All lines showing the Phi selective growth phenotype were genetically characterized via PCR and proved positive for the presence of the *ptxD* transgene sequence (nuclear and chloroplastic) (Figure 3).

To substantiate the observed putative selective growth advantage conferred by the mutagenized PTXD version, we set up a growth kinetics analysis in a laboratory-scale photobioreactor. To this end, we included two independent transplastomic lines for each PTXD version (TP *ptx*D WT and TP *ptx*D Opt), the untransformed T222 wild type strain and the nuclear transformant N *ptxD* WT. All transgenic lines were cultivated in TA-Phi (phosphite-supplemented) medium under continuous illumination with 200 μE light intensity and their growth monitored.

**Figure 4:**
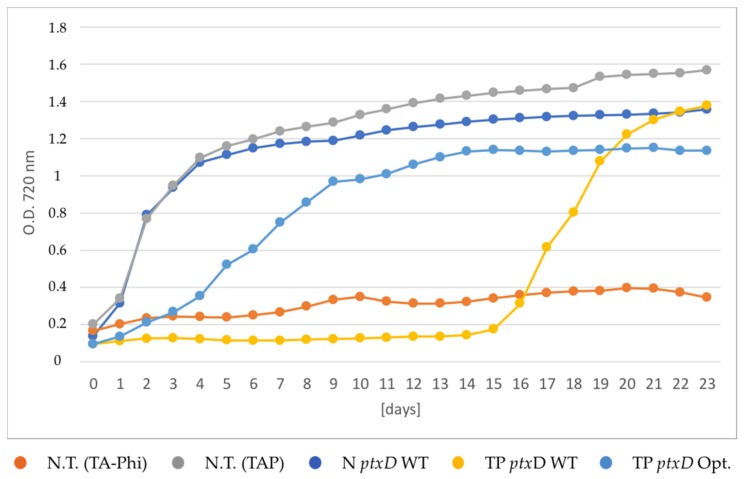
Growth curves of transgenic lines expressing the *ptxD* transgene in different cellular compartments. The selective growth phenotype was assessed by cultivating the algae in TA-Phi medium, following a phosphate-depletion treatment. The untransformed T222 wild type was cultivated in both TA-Phi (red trace) and TAP (grey trace) media as negative and positive controls, respectively. The nuclear transformant N *ptxD* WT (dark blue trace) and two independent transplastomic lines carrying the two PTXD versions (TP *ptxD* WT, yellow trace and TP *ptxD* Opt, light blue trace, respectively) were included in the experiment. Traces refer to the average of two technical replicates of growths performed in parallel. Cell density was measured by recording optical density at 720 nm.

A positive and a negative control, consisting of the untransformed T222 wild type strain cultivated in TAP (phosphate supplemented) and TA-Phi medium, respectively, were included. With this experimental set-up we could reproduce the previously inferred differences in the selective growth performance between lines. As shown in Figure 4, the N *ptxD* WT line grew in TAPhi medium (phosphite-supplemented) at a similar rate as the untransformed T222 wild type strain does in TAP medium (phosphate-supplemented).

This observation indicates that the availability of phosphite is not limiting in the cytoplasmic compartment of the alga and that the amount of nuclear-encoded recombinant PTXD enzyme is sufficient to efficiently catalyze the conversion of phosphite into phosphate by using the available NAD^+^ cytosolic pool. The inability to grow in TA-Phi medium displayed by the untransformed algal strain demonstrates that the PTXD activity is strictly required for the Phi growth phenotype. The TP *ptx*D Opt transplastomic lines displayed an intermediate growth phenotype, but this was significantly faster than that of the TP *ptx*D WT transplastomic line expressing the wild type PTXD enzyme. The latter displayed the slowest growth phenotype, reaching the plateau phase about 14 days later than the strain carrying the mutagenized PTXD counterpart, and 16 days after the PTXD WT nuclear transformant.

### 2.3. Faster Selective Growth is Related to a Higher Catalytic Efficiency of the Mutagenized PTXD Version

**Figure 5:**
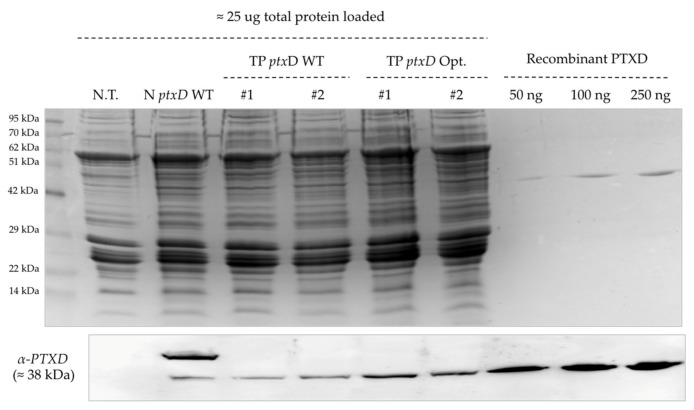
Biochemical characterization of produced transgenic lines displaying phosphite-metabolizing activity. A western blot analysis using the anti-PTXD antibody showed comparable amounts of recombinant enzyme expressed in the transplastomic lines carrying the two PTXD versions. The nuclear transformant N ptxD WT, the untransformed T222 wild type and variable amounts of the recombinant protein expressed in *E. coli* were included as controls. The upper panel displays a Coomassie-stained SDS-PAGE gel confirming equal amounts of total proteins for all lines (25 μg). Densitometric analysis yielded a relative intensity of the 29 kDa band of 1:1.8:1.7:1.1:2.1:1.9 for, respectively, lanes N.T, N*ptxD* WT, #1,#2,#1,#2 (from left to right).

We next characterized biochemically all transgenic lines via western blotting using the anti-PTXD antibody to assess the levels of recombinant enzyme. For this determination, we loaded equal amounts of total algal protein extracts based on Bradford’s assay and further normalized the loading of each lane base of densitometry of the Coomassie-stained gel (Figure 5). Upon normalization, we could not observe striking differences in the levels of PTXD protein among the lines, suggesting that the growth advantage displayed by the TP *ptx*D Opt lines with respect to TP *ptx*D WT transplastomic lines was derived from a superior catalytic efficiency of this modified enzyme version rather than from a higher level of PTXD in the plastid. An unexpected higher molecular weight band was observed in the N *ptxD* WT nuclear transformant, which could correspond to a highly glycosylated form of the enzyme, and was observed in a number of independent strains of *Chlamydomonas* upon nuclear transformation (not shown).

### 2.4. The Optimized PTXD Version Enables Fast and Reliable Recovery of Transformants

**Figure 6:**
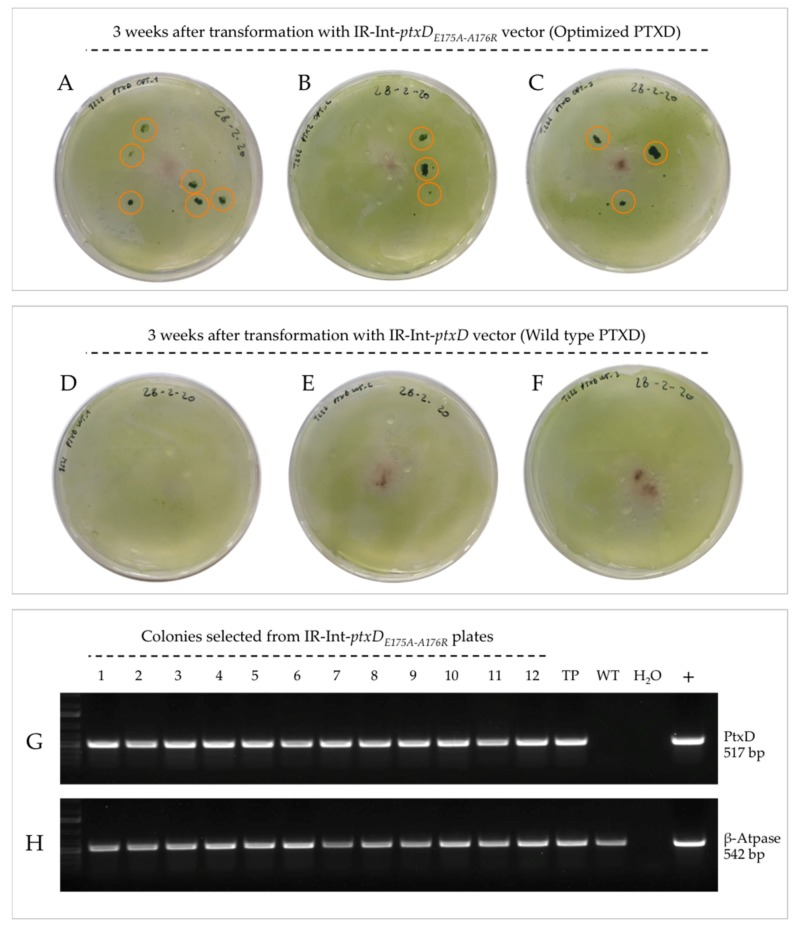
PTXD-based genetic transformation of the chloroplast genome coupled to phosphite metabolism-based selection. One-week phosphorous-starved T222 wild type cells were transformed with the IR-int vectors carrying the two PTXD versions. After 3 weeks, a number of colonies were clearly distinguishable on independent transformation plates for the Int-*ptxD_E175A-A176R_* vector (panel **A**, **B** and **C**, orange circled). No colonies could be retrieved following transformation with the wild type enzyme version (panel **D**, **E** and **F**). True transformation events were confirmed for all selected colonies via PCR-based genetic analysis on genomic DNA targeting the PTXD transgene (1-12, panel **G**). A positive reaction against the plastid ATP-ase β subunit gene was included (panel **H**). TP refers to characterized transplastomic PTXD lines produced in this work; WT is the untransformed T222 wild type control.

We next investigated whether the modified PTXD enzyme version could serve as a reliable selectable marker for the genetic transformation of the algal plastome using a phosphite metabolism-based selection strategy. To this end, we transformed, in parallel, three batches of one-week phosphorous-starved cells with the IR-vectors carrying the wild type and modified PTXD version on TA-Phi (1 mM) agar plates, as previously described [18]. As shown in Figure 6, the transformation with the Int-*ptxD_E175A-A176R_* vector resulted in the formation of colonies three weeks post-bombardment (panels A, B and C), while no colonies were visible when the wild type PTXD version was used for transformation (panels D, E and F). In the case of IR-Int-*ptxD_E175A-A176R_,* 12 large colonies were assessed via genetic analysis. The PCR analysis confirmed the presence of the PTXD transgene in all of them, confirming their identity as true transformation events (Figure 6, panel G). These results demonstrate that the modified PTXD *ptxD_E175A-A176R_* mutant is a superior selectable marker gene compared to the native enzyme.

The optimized PTXD version enabled a reliable recovery of transformants, reducing the selection times from 60 days [21] to three weeks and virtually abolishing the risk of false positives that could follow the unspecific background algal growth sustained by the released polyphosphates of untransformed dead cells. In fact, that selective growth phenotype conferred by the optimized enzyme is already present at early stages post-bombardment, when a low transgene copy number is expected to be present in the transformed transplastomic cells.

## 3. Discussion

To date, chloroplast transformation approaches in *C. reinhardtii* rely on the use of antibiotics as selective agents and cognate resistance genes. The *aadA* gene, encoding an aminoglycoside-adenyltransferase that inactivates spectinomycin [22] constitutes the “gold-standard” selectable marker for chloroplast transgenesis in this species. Other protocols, such as those based on the recovery of photoautotrophy using non-photosynthetic, acetate-requiring recipient mutant strains, have certain advantages but involve time-consuming selection protocols [24]. The use of antibiotic resistance genes is generally discouraged because of concerns over potential transgene escape in the environment and horizontal transfer to harmful pathogens [25]. Nonetheless, from a pure biotechnological perspective, this type of selection strategy is problematic, since the genetic instability of transgenic organisms can manifest when the selective agent is omitted during large-scale cultivation. This is particularly relevant in the case of the polyploid algal plastome, as transgenes can be rapidly lost due to segregation following the relaxation of selective pressure if the homoplasmic condition was not fully reached. Given these premises, there is an urgent need to abandon the use of antibiotics in the field of transplastomic algal biotechnology and to foster the development of alternative selective growth strategies which do not involve the use of potentially toxic compounds. In this respect, the PTXD transgene holds a huge potential with still unexplored applications, and it is likely that the current PTXD system can be improved. Indeed, the expression of the PTXD enzyme in the plastid serves two highly desirable purposes: it functions as a growth selector enabling transgenic microalgae to grow in non-sterile conditions reducing the risk of culture contamination, and it also represents an environmentally friendly selectable marker for plastome engineering. This approach truly represents a pioneering research field, with only a few reports having addressed this topic so far [16,17,18]. Nevertheless, the function of the PTXD enzyme in the chloroplast appears to suffer from some limitations [21], possibly connected with this peculiar cellular environment, that hinders its catalytic activity. In principle, the pH and temperature fluctuations that occur within the chloroplast stroma are compatible with the optimum working range of the enzyme [26]. Instead, more plausible physiological constraints for the enzyme activity are possibly related to the availability of its native cofactor NAD^+^ and the abundance of its substrate, phosphite, in the plastid. This latter factor does not constitute a limitation, since phosphite and phosphate can be handled by the same type of transporters in biological systems [27] and are thus imported into the chloroplast via the same antiporter system in exchange with triose phosphates [28]. Instead, we reasoned that the amount and the redox status of NAD^+^, the preferential cofactor of the PTXD enzyme [14], are likely the main physiological parameters affecting the enzyme activity. Indeed, the supply of NAD^+^ to the chloroplast relies on its unidirectional import from the cytoplasm, which, in turn, represents the only site of the *de novo* synthesis of this cofactor in the cell [29]. In addition, this pathway constitutes a starting point for the NAD kinase-mediated phosphorylation of NAD^+^ [30] to produce NADP^+^, the latter being the dominant nicotinamide adenine dinucleotide cofactor found inside the chloroplast. Although NADP^+^ can substitute NAD^+^ in vitro for the PTXD-catalyzed conversion of phosphite into phosphate, the overall efficiency of this reaction is significantly lower compared with the one in presence of NAD^+^ [14].

Nonetheless, the plastid is preferentially enriched in the reduced form of NADP^+^, NADPH, as the light-dependent reactions of photosynthesis occurring on the thylakoid membrane system use NADP^+^ as the terminal electron acceptor. A dynamic redox interconversion between NADP^+^ and NADPH occurs in the chloroplast as a consequence of the recycling of this cofactor between the light-dependent photosynthetic reactions and the Calvin cycle [31]; yet, the supply of regenerated NADP^+^ is likely not sufficient to sustain efficient activity of the PTXD enzyme. As a result, the overall amounts of the preferential and auxiliary cofactors of the PTXD enzyme probably restricts its function, leading to suboptimal growth performance of the alga. The delayed growth of transplastomic lines carrying the wild type PTXD version can be explained by a sustained phosphate starvation response continuing in the presence of phosphite [32,33].

*C. reinhardtii*’s main phosphate stores consist of the nucleic acids making up the nuclear and organellar genomes and the so called cytosolic polyphosphate bodies [34], along with some minor stores in the cell wall [35]. Under severe phosphate starvation, it can be envisaged that intracellular phosphate stores are mobilized to enable basal cell metabolism, involving a reduction in the number of copies of the plastid genome [36]. This situation would produce a quiescent state during which the algae will not divide. Once a sufficient amount of free phosphate ion is made available in the cell from these stores, and from the constrained activity of the PTXD enzyme, cell division can resume. This could explain our observation that the *C. reinhardtii* cell expressing the WT PTXD in the chloroplast showed a delay in reaching the exponential growth phase regarding the cell expressing the PTXD in the cytoplasm.

For these reasons, we have rationally mutagenized the PTXD enzyme in order to improve its catalytic efficiency within the chloroplast of *C. reinhardtii.* The substitution of two adjacent amino acid residues localized in the cofactor-binding pocket of the enzyme enabled the use of both nicotinamide adenine pools (NAD^+^ and NADP^+^) by PTXD. The mutagenized PTXD version possesses a higher turnover number resulting in faster phosphite-processing activity leading to increased availability of inorganic phosphate in the organelle. In this situation, the photosynthesis-dependent, phosphate-requiring carbon metabolism [37] can be sustained more efficiently, ultimately leading to a higher synthesis of sugars and starch reserves compared to the physiologically-constrained wild type enzyme, ultimately favoring a faster selective growth of the algae in nutrient (phosphate)-limited conditions. This notion is in line with the faster growth in phosphite media of the *C. reinhardtii* cells expressing the mutagenized PTXD in their chloroplasts as compared to those expressing the WT PTXD in the chloroplast.

**Figure 7:**
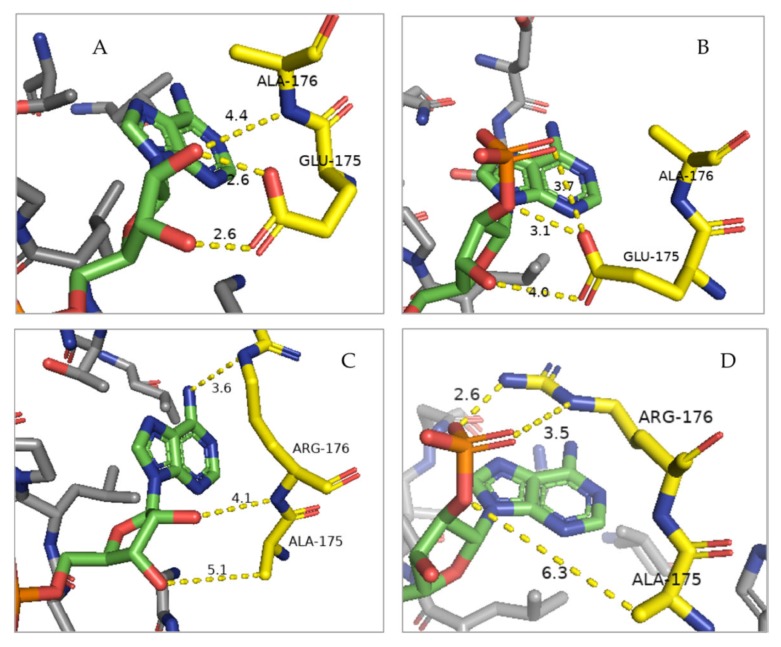
Molecular model of the cofactor-binding pocket in the PTXD enzyme showing the effects of the double amino acid substitution (E175A, A176R) on the docking of the NAD^+^ vs NADP^+^ cofactors. The interactions between the nucleotide moiety of both cofactors (in green) with residues 175 and 176 (in yellow) are shown. Charge interactions are depicted as yellow dashed lines with distances in Angstrom between the hydrogen bond acceptor atoms to hydrogen atoms. (**A**) NAD^+^ cofactor binding by the wild type enzyme, where multiple polar interactions are allowed between the 2′- and 3′-hydroxyl groups (in red) of the nucleotide and the side chain of glutamate-175 and the peptidyl nitrogen of residue alanine-176. (**B**) NADP^+^ binding by the wild type enzyme. This is a suboptimal interaction due to the charge repulsion/steric hindrance effect from the negatively charged carboxyl group of glutamate-175, which prevents docking. This is consistent with the observed lower K_M_ for NADP^+^ [4]. (**C**) Binding of NAD^+^ by the mutagenized PTXD. Interactions between cofactor and protein are similar to the case of WT since, following the replacement of residue 175 with the smaller side chain of alanine, the distances are only slightly wider allowing for a sterical relaxation of the local charge environment. (**D**) Binding of NADP^+^ to the mutagenized enzyme. The substitution of glutamate-175 to alanine enables docking of the 2′-phosphate cofactor group, otherwise physically constrained. Moreover, the alanine-176 to arginine replacement results in a stabilization of the phosphate moiety of the cofactor via compatible ionic and polar interactions. Structural analysis was performed with PyMol software using previously published structures [38].

From a mechanistic perspective, the introduced double amino acid substitution is assumed to affect the charge distribution around the cofactor-binding pocket, creating an environment that favors the simultaneous docking of both NAD^+^ and NADP^+^ cofactors, while causing only a minor structural alteration of the overall protein topology [38]. The negatively charged phosphate moiety of the NADP^+^ molecule can be more easily accommodated in the pocket as a result of the neutralization of the negative charge of the native residue glutamate-175 via its substitution with alanine and the stabilization of the interaction promoted by the introduction of the positively charged residue arginine in place of alanine-176 (Figure 7).

Altogether, our results support the usefulness of the PTXD enzyme as an efficient system for algal biotechnological applications. The optimized enzyme version enables faster selective growth of transplastomic algae in nutrient (phosphate)-limited conditions, thus allowing this system to be implemented in large-scale cultivation. In addition, the modified PTXD transgene favors a faster and more reliable recovery of transplastomic transformants, fully establishing this system an alternative, environmentally friendly selection method that will replace antibiotic resistance gene-based protocols.

## 4. Materials and Methods

### 4.1. Algal Strains and Cultivation Strategies

*C. reinhardtii* wild type strain T222+ (CC-5101) [39] was obtained from the Chlamydomonas Resource Center and used as recipient strain for all genetic modifications described in this work. Growth experiments were conducted in a laboratory-scale photobioreactor (Multi-Cultivator MC 100-OD, Photon System Instruments, Drasov, Czech Republic) in mixotrophic conditions using an acetate-supplemented tris-acetate-phosphate rich medium (TAP) [40] under the following controlled conditions: continuous light at 200 μmol photons m^−2^ s^−1^ irradiance, 22 °C and constant air bubbling. For general purposes, algae were propagated on TAP-agar (1,5 % *w*/*v*) plates containing appropriate antibiotics. Selective growth experiments in phosphite were conducted by substituting the phosphorous source sodium phosphate (K_2_PO_4_) with sodium phosphite (Na_2_HPO_3_ 5H_2_O_,_ Sigma Aldrich), while maintaining the same ion concentration. Phosphite-containing rich and minimal media are hereinafter referred to as TA-Phi and HS-Phi, respectively. A precultivation step in TAP medium preceded the selective growth experiments until a density of 10^6^ cells/mL was reached. Cultures were subsequently pelleted and re-suspended and cultivated in TA medium (phosphorous devoid) for 4 days to exhaust intracellular phosphate stores (phosphate depletion) before being exchanged to TA-Phi medium.

### 4.2. Transformation of the Chloroplast Genome of C. reinhardtii

The *ptxD* gene sequence, coding for the phosphite dehydrogenase enzyme of *Pseudomonas stutzeri* WM88, was obtained from the UniProt database (UniProtKB—O69054), optimized according to the AT-rich codon bias of the chloroplast genome of *C. reinhardtii* [41] using the online tool OPTIMIZER [42] and obtained using GeneScript (Leiden, Netherlands) as a synthetic gene in the pUC57 vector flanked by *NcoI* and *SphI* restriction sites. Nucleotide sequence of the chloroplast codon usage-optimized phosphite dehydrogenase (ptxD) gene sequence can be found in Supplementary. The mutagenized version of the PTXD enzyme, carrying the substitution of the two adjacent residues Glu-175 and Ala-176 with Ala and Arg, respectively (hereinafter referred to as *ptxD_E175A-A176R_*) was produced via site directed mutagenesis of the wild type *ptxD* sequence contained in the pUC57 vector with a pair of mismatch-containing primers (Ptxd_E175A_A176R FW TTGTTCTGTTTGTGTATCTAAAGCTTTACGAGCATGATATTGTAATGTAGCACCCCAACC; Ptxd_E175A_A176R_RV GGTTGGGGTGCTACATTACAATATCATGCTCGTAAAGCT TTAGATACACAAACAGAACAA) using the Quick Change Lightning Site-Directed Mutagenesis Kit (Agilent Technologies, Santa Clara, USA) following the manufacturer’s instructions. The plasmid used for the chloroplast transformation of the wild type T222 strain is the IR-int vector [21]. This vector contains two homology regions within the inverted repeats of the chloroplast genome of *C. reinhardtii*, targeting the exon V of the *psbA* gene (D1 subunit of photosystem II) and the 5S ribosomal RNA and a portion of the 23S ribosomal RNA, respectively, enabling a homologous recombination-based transgene insertion at the BamHI site found between these two genomic loci. This vector contains cis-acting regulatory elements derived from endogenous chloroplast genes driving the expression of the transgenes of interest (*atpA* promoter and 5′ UTR) and to ensure the stability of its transcript (3′ UTR of the RuBisCo large subunit, *rbcl*), along with the selectable marker gene *aadA* [22], providing the detoxifying activity towards the antibiotic spectinomycin. In this work, we nevertheless opted for a different cis-acting element pair to drive the expression of the PTXD enzyme(s), derived from the AtpB-int vector [21] that is employed to transform the non-photosynthetic, ATP synthase β subunit-deficient *C. reinhardtii* strain *FUD50* (CC-1185). In particular, the AtpB-int vector provides the highly efficient *psaA* (subunit A of photosystem I core) promoter. To achieve such cassette configuration for our transgene, a two-step subcloning operation was performed starting from the pUC57 vector containing the *ptxD* sequence, first into the AtpB-int vector and finally into the IR-int vector. To this end, the wild type pUC57-*ptxD* construct and the herein created mutagenized pUC57-*ptxD_E175A-A176R_* version were excised via *NcoI* and *SphI* restriction enzymes from the pUC57 vector and firstly ligated into the AtpB-int vector [21], previously digested with the same restriction enzyme pair. By doing so, both PTXD versions were placed under the transcriptional control of the strong, plastid native cis-acting regulatory elements provided by this vector. These larger cassettes, including the PTXD coding sequence(s) and the newly acquired *PpsaA* and 3′ *rcbL*, cis-acting regulatory elements were subsequently excised via *ClaI* and *SmaI* restriction sites and ligated into the IR-int vector to produce the IR-Int:*ptxD* and IR-Int-*ptxD_E175A-A176R_*, intended to be used for the transformation of the chloroplast genome of the T222 (CC-5101) wild type *C. reinhardtii* strain. Transplastomic *C. reinhardtii* lines were created via the biolistics transformation method using the modified IR-int vectors following an established protocol [43] using plasmid DNA-covered 0.6 μm diameter gold microcarriers (Bio-Rad, Hercules, CA, USA) and a PDS-1000/He gene gun system (Bio-Rad). Transplastomic *C. reinhardtii* colonies were selected on spectinomycin-containing (100 μg/μL) TAP-agar plates and individual colonies were subsequently subcultured for several rounds (6-8) on selective plates in order to increase the transgene copy number until full homoplasmy. Chloroplast transformation of *C. reinhardtii* exploiting phosphite metabolism-based selection was performed following the method described above using one-week phosphorous-starved T222 wild type cells. Algae were plated on TA-Phi (1 mM) agar medium and transformed in parallel with the IR-int vectors carrying the two PTXD versions. Genetic characterization of putative transformants was performed via colony PCR directly from three-week-old colonies from the original transformation plates using primers specific for the chloroplast codon usage-optimized PTXD coding sequence (described in Section 4.5).

### 4.3. Transformation of the Nuclear Genome of C. reinhardtii

A nuclear transformant, expressing the PTXD enzyme in the cytoplasm, was created via electroporation of T222 *C. reinhardtii*, following a previously described standard transformation protocol, and served as additional control in all experiments described in this work. To this end, the pChlamy-4 vector (Life Technologies Corporation, Carlsbad, CA, USA), containing the intron-less *ptxD* sequence optimized for the codon usage of the nuclear genome of *C. reinhardtii* under the control of the *Hsp70A-RbcS2* chimeric constitutive promoter [15], was linearized with the *ScaI* restriction enzyme and 500 ng of the plasmid were used to transform *C. reinhardtii* cells. After 24 h recovery in the dark, cells were plated on TAP-agar plates supplemented with the selective agent antibiotic zeocin. Zeocin-resistant clones were subcultured, screened for their ability to grow in TA-Phi medium in the presence of phosphite as sole phosphorous source and further genetically and biochemically characterized.

### 4.4. Production of Recombinant PTXD Protein in E. coli

The PTXD enzyme was expressed as a recombinant protein in *E. coli* to be used as antigen for the production of an anti-PTXD specific polyclonal antibody. To this end, the *ptxD* gene sequence optimized for nuclear codon usage of *C. reinhardtii* was amplified via PCR from the pChlamy-4 vector [15] with primers providing restriction sites *NdeI* and *BamHI*.

The *ptxD* cassette flanked by restriction sites was subsequently introduced into the multiple cloning site of the pET28a+ vector (Novagen) via digestion and ligation to express a recombinant PTXD protein carrying an N-teminal polyhistidine (6x) tail. The obtained plasmid pET28a+*ptxD-His-tagged* was transformed into BL21 (DE3) pLysS *E. coli* electrocompetent cells, and protein overexpression was induced by the addition of isopropyl β-d-1-thiogalactopyranoside (IPTG, 0.1 mM) and culturing of cells for 16 h at 16 °C. Next, the recombinant His-tagged PTXD protein was purified via affinity chromatography on gravity columns packed with a nickel-charged sepharose resin (GE Healthcare, Life Sciences, Pittsburgh, PA, USA). To this end, the crude bacterial lysate was loaded on the column followed by extensive washing in the presence of 20 mM imidazole. The soluble target protein was then eluted with 500 mM imidazole and 4 elution fractions were pooled, buffer exchanged with PD-10 desalting columns (GE-Healthcare) and concentrated using Amicon centrifugal filters (Merk Millipore). The recombinantly expressed PTXD protein was checked for purity on Coomassie-stained SDS-PAGE gels, and approximately 2 mg of protein were used for rabbit immunization and production of the polyclonal antibody (Davids Biotechnologies, Regensburg, Germany).

### 4.5. Phenotypical, Genetic and Biochemical Characterization of All Transgenic Lines

All produced transgenic lines underwent an initial phenotypical screening to assess their ability to selectively grow in a phosphate-devoid, phosphite-supplemented medium. One nuclear transformant and two independent transplastomic lines for each PTXD version were selected for further characterization. A PCR-based genotyping analysis was performed on genomic DNA extracted with a quick protocol [44] to detect the presence of the *ptxD* transgene sequence(s). For the nuclear transformant a specific primer pair (Ptxd_596_F CCTCGTCCGACTTCATCCTG and Ptxd_805_R CCCAGTCCTCCATCTCGAAG) was employed to amplify a 210 bp sequence. For the transplastomic lines, a primer pair specific for the chloroplast codon usage-optimized *ptxD* version was used instead, giving a 517 bp amplicon (chl_PTXD_CDS_FW CCAAAATTAGTTATTACACATCGTG and chl_PTXD_CDS_RV CATGATATTGTAATGTA GCACCC). Positive control reactions were conducted with primers specific for the plastid ATP-ase β subunit gene producing a 542 bp amplicon (atpB_CDS_FW GTAAATACTTCAGCTACGAAGAATG and atpB_CDS_RV ATGTTAACAAACAAGACGTATTATTCT).

Selected lines were further characterized biochemically for the expression of the recombinant PTXD protein via western blotting experiments using the custom-made anti-PTXD antibody. To this end, total algal proteins were extracted using an established protocol [45] starting from 15 mL of a *C. reinhardtii* culture from early exponential phase. Briefly, cells were harvested using centrifugation and the pellet was re-suspended in 300 μL of solution A (0.1% Na_2_CO_3_), 200 μL of solution B (5% SDS, 30% sucrose) and 25 μL of β-mercaptoethanol. Samples were incubated at room temperature for 25 min under gentle agitation. Approximately 25 μg of total proteins were separated on denaturating SDS-PAGE gels followed by Coomassie staining. In parallel, SDS-PAGE-separated protein samples were transferred to a nitrocellulose membrane using a single buffer device (Bio-Rad) to proceed with the immunodecoration. Blotted membranes were incubated with blocking buffer (PBS 1X, 5% milk powder, 2% TWEEN) and subsequently incubated overnight at 4 °C with the anti-PTXD antibody. Following washes, membranes were incubated with a secondary antibody coupled to a horseradish peroxidase enzyme, and signal acquisition was performed with the enhanced chemiluminescence (ECL) method using a ChemiDoc (Bio-Rad) imaging system.

### 4.6. Bioinformatics Methods

Bioinformatics analyses and protein structure modelling were performed with the PyMOL Molecular Graphics System, Version 2.0 Schrödinger, LLC. (https://www.pymol.org/) using the previously created [38] Protein Data Bank (PDB) files of PTXD structures 4E5N, 4E5P and 4E5M, obtained from the PDB databank.

## 5. Conclusions

The optimized version of the PTXD enzyme constitutes a methodological advance in the field of genetic transformation of the chloroplast genome of *C. reinhardtii*, since it favors two highly desirable features in algal biotechnology. On the one hand, it strengthens the reliability of using the *ptxD* transgene-phosphite fertilization approach as a feasible strategy to prevent culture contamination in non-sterile conditions without compromising on algal growth performance and biomass accumulation. Moreover, the rationally improved PTXD version enables the reliable and efficient use of this newly introduced selectable marker both in basic and applied strategies that pursue safe approaches to transformation of the chloroplast genome.

## Supplementary Materials

The following information is available online at https://www.mdpi.com/2223-7747/9/4/473/s1. Nucleotide sequence of the chloroplast codon usage-optimized phosphite dehydrogenase (ptxD) gene sequence.
