# Supplementary material for: A Phosphite Dehydrogenase Variant with Promiscuous Access to Nicotinamide Cofactor Pools Sustains Fast Phosphite-Dependent Growth of Transplastomic Chlamydomonas reinhardtii
Source: Plants (Basel). 2020 Apr 8;9(4):473. doi: 10.3390/plants9040473 (PMC7238262; doi:10.3390/plants9040473)
Supplement: Supplementary file 1 [file plants-09-00473-s001.pdf]

Nucleotide sequence of the chloroplast codon usage-optimized phosphite dehydrogenase (*ptxD*) gene sequence. In green are highlighted the nucleotides that were exchanged via site-directed mutagenesis to create the E175A/A176R double amino acid substitution.

ATGTTACCAAAATTAGTTATTACACATCGTGTTCATGATGAAATTTTACAATTATTAGC
TCCACATTGTGAATTAATGACAAATCAAACAGATTCAACATTAACACGTGAAGAAAT
TTTACGTCGTTGTCGTGATGCTCAAGCTATGATGGCTTTTATGCCAGATCGTGTTGATG
CTGATTTTTTACAAGCTTGTCCAGAATTACGTGTTGTTGGTTGTGCTTTAAAAGGTTTT
GATAATTTTGATGTTGATGCTTGTACAGCTCGTGGTGTTTGGTTAACATTTGTTCCAGA
TTTATTAACAGTTCCAACAGCTGAATTAGCTATTGGTTTAGCTGTTGGTTTAGGTCGTC
ATTTACGTGCTGCTGATGCTTTTGTTCGTTCAGGTGAATTTCAAGGTTGGCAACCACAA
TTTTATGGTACAGGTTTAGATAATGCTACAGTTGGTATTTTAGGTATGGGTGCTATTGG
TTTAGCTATGGCTGATCGTTTACAAGGTTGGGGTGCTACATTACAATATCATGAAGCT
AAAGCTTTAGATACACAAACAGAACAACGTTTAGGTTTACGTCAAGTTGCTTGTTCAG
AATTATTTGCTTCATCAGATTTTATTTTATTAGCTTTACCATTAAATGCTGATACACAA
CATTTAGTTAATGCTGAATTATTAGCTTTAGTTCGTCCAGGTGCTTTATTAGTTAATCC
ATGTCGTGGTTCAGTTGTTGATGAAGCTGCTGTTTTAGCTGCTTTAGAACGTGGTCAAT
TAGGTGGTTATGCTGCTGATGTTTTTGAAATGGAAGATTGGGCTCGTGCTGATCGTCC
ACGTTTAATTGATCCAGCTTTATTAGCTCATCCAAATACATTATTTACACCACATATTG
GTTCAGCTGTTCGTGCTGTTCGTTTAGAAATTGAACGTTGTGCTGCTCAAAATATTATT
CAAGTTTTAGCTGGTGCTCGTCCAATTAATGCTGCTAATCGTTTACCAAAAGCTGAAC
CAGCTGCTTGTTAA
